# Higher-Order Cu^I^-Based Cages via Subcomponent Self-Assembly

**DOI:** 10.1021/acs.accounts.5c00081

**Published:** 2025-03-25

**Authors:** Huangtianzhi Zhu, Natasha M. A. Speakman, Tanya K. Ronson, Jonathan R. Nitschke

**Affiliations:** Yusuf Hamied Department of Chemistry, University of Cambridge, Lensfield Road, Cambridge CB2 1EW, United Kingdom

## Abstract

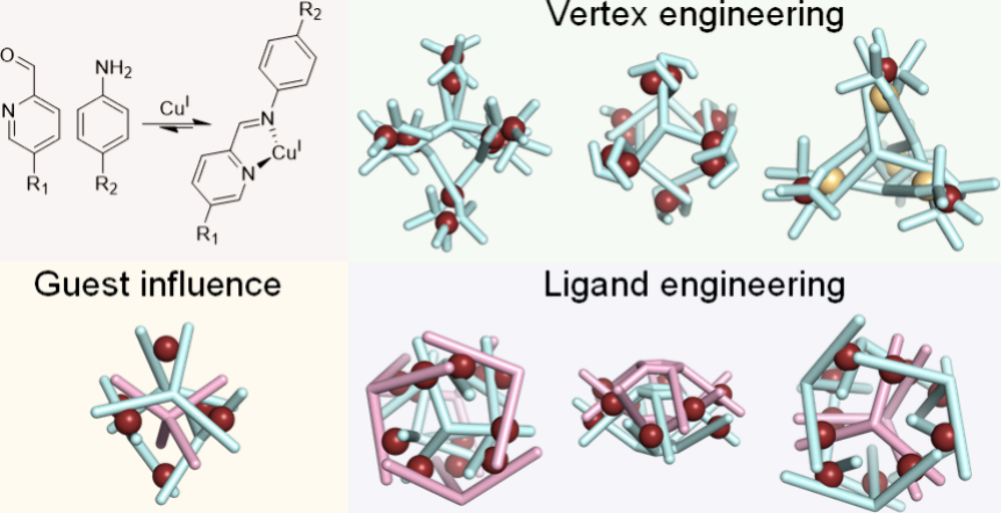

Coordination cages formed via subcomponent self-assembly have found applications in fields including separation, sensing, catalysis, and the stabilization of reactive species, due to their guest binding abilities. Subcomponent self-assembly, which combines dynamic covalent bond (C=N) formation and reversible metal coordination (N→Metal), has enabled the preparation of many intricate polyhedral structures with minimal synthetic effort. This method has been used to prepare multitopic pyridyl-imine ligands that form the edges or faces of polyhedra, with octahedral metal ions, including Fe^II^, Co^II^, and Zn^II^, defining the vertices. The use of Cu^I^ in subcomponent self-assembly is less widely reported, as the tetrahedral coordination geometry of Cu^I^ requires only two bidentate ligands, which can lead to lower-nuclearity assemblies instead of three-dimensional cages. The coordination flexibility of Cu^I^ also adds a challenge to the fabrication of well-defined nanostructures. This Account summarizes a series of higher-order Cu^I^-based coordination cages and the design principles derived from their syntheses. Starting with the development of Cu^I^ assemblies and the challenges of preparing Cu^I^ cages, we discuss the circumvention of oligomer formation and control of the self-assembly process with Cu^I^ through (i) ligand engineering, (ii) vertex design, and (iii) guest-induced structural transformations. Aromatic stacking between corranulene-containing ligands is exploited to produce a 5-fold interlocked [2]catenane, whereas the incorporation of a sterically hindered triptycene subcomponent that minimizes aromatic stacking produces a double-octahedron and a hexagonal prism. These structures illustrate the importance of ligand engineering for obtaining complex Cu^I^ structures. We also explored the formation of cages with homo- or heterobimetallic vertices via two distinct strategies. First, dicopper(I) helicates were employed as cage vertices, and second, subcomponents with nonconverging coordination vectors were used. Such bimetallic vertices are challenging to incorporate when octahedral metal templates are used, but the flexibility of Cu^I^ renders them accessible. The closed-shell electronic configuration of Cu^I^ can endow the cages with photoluminescence, providing circularly polarized luminescence in the presence of helicity-enriched dicopper(I) vertices. The flexible coordination sphere of Cu^I^ also facilitates structural transformations upon the addition of suitable guests. One such system is able to self-sort to express the most thermodynamically stable host–guest complex and also undergo structural changes in response to different temperatures and solvents. The insights gained about the structural bases of these Cu^I^ cages may help enable the design of other novel Cu^I^ nanostructures with functions that may usefully differ from cages that exclusively incorporate octahedral metal centers.

## Key References

- RonsonT. K.; WangY.; BaldridgeK.; SiegelJ. S.; NitschkeJ. R.An *S*_10_-Symmetric 5-Fold Interlocked [2]Catenane. J. Am. Chem. Soc.2020, 142, 10267–1027232453562 10.1021/jacs.0c03349PMC7291353.^1^*A corannulene subcomponent that can undergo aromatic stacking is employed to construct a 5-fold interlocked Cu*^*I*^*[2]catenane, emphasizing the importance of dispersion interactions to drive structure formation*.
- ZhuH.; RonsonT. K.; WuK.; NitschkeJ. R.Steric and Geometrical Frustration Generate Two Higher-Order Cu^I^_12_L_8_ Assemblies from a Triaminotriptycene Subcomponent. J. Am. Chem. Soc.2024, 146, 2370–2378.38251968 10.1021/jacs.3c09547PMC10835662(2)*The unique geometry of triptycene enables the formation of two distinct Cu*^*I*^_12_*L*_8_*assemblies with T and D*_3_*point symmetries, respectively. Solvent and electronic effects also play key roles during their assembly*.
- ZhuH.; PesceL.; ChowdhuryR.; XueW.; WuK.; RonsonT. K.; FriendR. H.; PavanG. M.; NitschkeJ. R.Stereocontrolled Self-Assembly of a Helicate-Bridged Cu^I^_12_L_4_ Cage That Emits Circularly Polarized Light. J. Am. Chem. Soc.2024, 146, 2379–2386.38251985 10.1021/jacs.3c11321PMC10835658(3)*A Cu*^*I*^*cage with dicopper(I) helicate corners is prepared, whose helicity is controlled by chiral additives. The P- or M-biased cages exhibit chiroptical properties*.
- CarpenterJ. P.; RonsonT. K.; RizzutoF. J.; HeliotT.; GriceP.; NitschkeJ. R.Incorporation of a Phosphino(pyridine) Subcomponent Enables the Formation of Cages with Homobimetallic and Heterobimetallic Vertices. J. Am. Chem. Soc.2022, 144, 8467–8473.35511929 10.1021/jacs.2c02261PMC9121369(4)*Cages with homobimetallic Cu*^*I*^_2_*and heterobimetallic Cd*^*II*^*Cu*^*I*^*vertices are generated through the use of a phosphino(pyridine) subcomponent that exhibits different binding preferences arising from its phosphorus and nitrogen atoms*.
- SpeakmanN. M. A.; HeardA. W.; NitschkeJ. R.A Cu^I^_6_L_4_ Cage Dynamically Reconfigures to Form Suit[4]anes and Selectively Bind Fluorinated Steroids. J. Am. Chem. Soc.2024, 146, 10234–10239.38578086 10.1021/jacs.4c00257PMC11027141(5)*Tetrahedral guests drive the formation of mechanically bound suit[4]anes with formation of a T-symmetric Cu*^*I*^*pseudo-octahedral cage from a dynamic mixture of cage diastereomers*.

## Introduction

Coordination-driven self-assembly allows the construction of well-defined hollow metal–organic structures that have found applications in fields that include absorption and separation,^6,7^ drug delivery,^8,9^ luminescence,^10,11^ and molecular devices.^12,13^ These assemblies can be prepared using the technique of subcomponent self-assembly, whereby dynamic covalent (commonly C=N) and coordination (N→Metal) bonds are formed *in situ*.^14^ This technique has employed transition metal templates and pyridyl-imine ligands to form tetrahedra,^15^ cubes,^16^ and larger polyhedra.^17,18^ Most of the transition metal templates used have octahedral coordination geometries, which bring three pyridyl-imine ligands together into a tightly constrained configuration around each metal center.^19−21^ Cu^I^, however, has seen less use, with most reports involving linear^22−24^ and circular^25−27^ helicates. The intrinsic coordination flexibility of Cu^I^ and less constrained junction formed by only two bidentate ligands add challenge to the generation of more complex self-assembled structures incorporating Cu^I^. Studies employing preformed organic linkers in the Schmittel and Lehn groups demonstrated that careful ligand design for heteroleptic complex formation are required to generate grids,^28−30^ ladders,^31^ racks,^32,33^ and cylindrical nanostructures.^34,35^ Other structures involving Cu^I^ and polytopic pyridyl-imine ligands have resulted in the formation of simpler grids, helicates, and circular helicates.^22,23,25,36^

Copper(I) structures exhibit useful properties, such as photoluminescence,^37,38^ redox-driven transformation between Cu^I^ and Cu^II^,^39−41^ and stability in aqueous media,^42^ offering potential for interactions with biomolecules. These characteristics make Cu^I^-based cages particularly attractive for a wide range of applications. Compared with cages that incorporate other metals, Cu^I^ complexes often exhibit strong metal-to-ligand charge transfer bands, which can be modulated through ligand design, enabling the construction of systems with desirable optical properties. The redox activity of Cu^I^ facilitates reversible transformations between oxidation states, making Cu^I^-based cages promising candidates for stimuli-responsive materials and photoredox catalysts.^43^ Cu^I^-based cages also demonstrate relatively high structural diversity and tunability due to the flexible coordination geometry. This adaptability enables the formation of cage structures with widely varying cavity shapes, which allow precise control over guest encapsulation, selectivity, and reactivity.

This Account summarizes our recent work on subcomponent self-assembly with Cu^I^ to generate well-defined nanostructures. Our endeavors to control the self-assembly process and obtain intricate architectures have principally focused upon ligand design and vertex engineering. The ability of guests to template the formation of symmetric cages and induce structural transformations is also discussed.

## From Mononuclear Copper(I) Complexes to Three-Dimensional Cages

**Figure 1:**
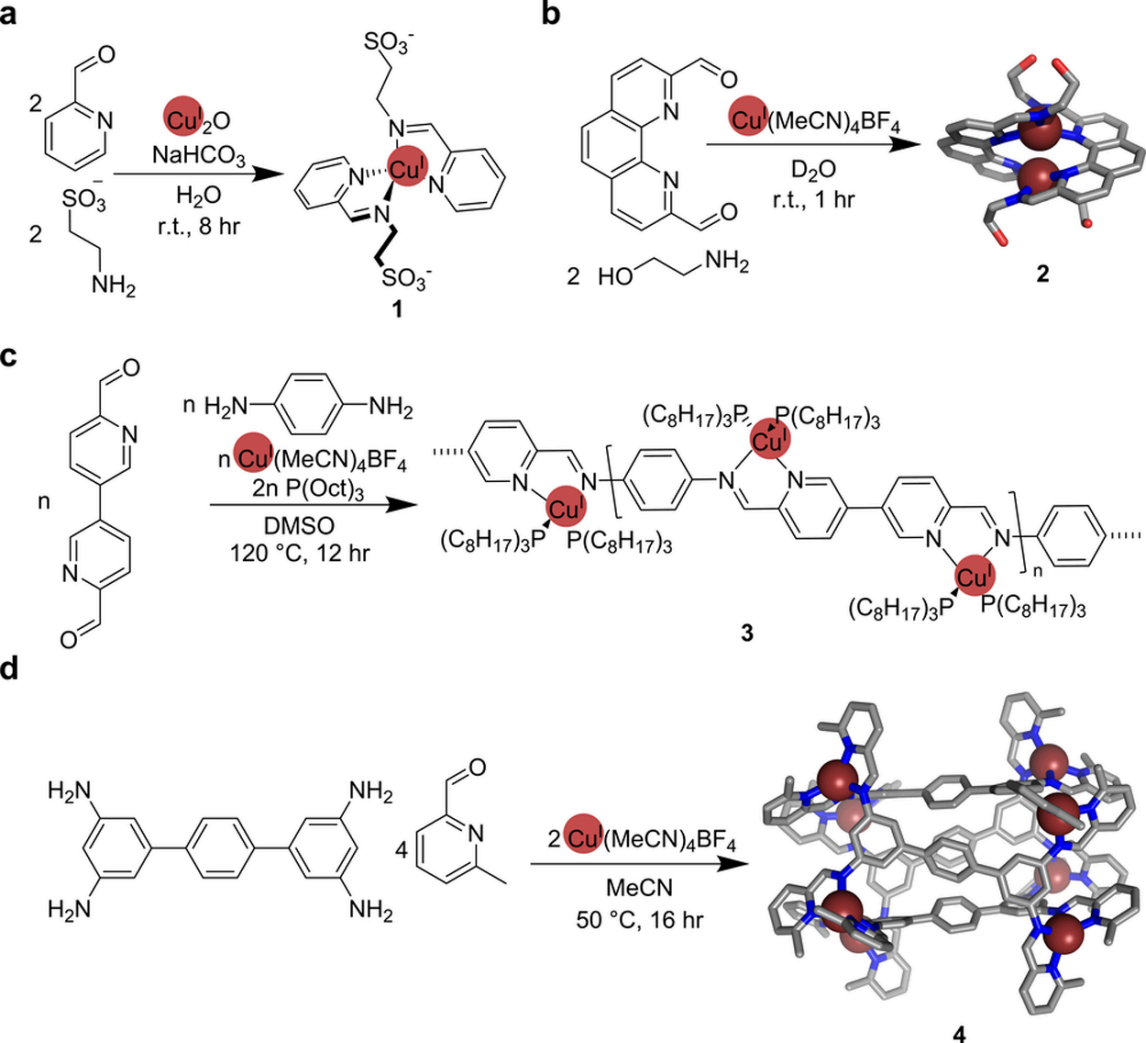
(a) Formation of the mononuclear Cu^I^ complex **1**. (b) Synthesis and crystal structure of dicopper(I) helicate **2**. (c) Formation of single-stranded metallopolymer **3**. (d) Synthesis and crystal structure of Cu^I^_8_L_4_ tube **4**.

Our interest in Cu^I^ was spurred by the discovery that Cu^I^ could template imine formation in aqueous solution to form a stable mononuclear complex.^42^ The reaction between taurine, 2-formylpyridine, and Cu^I^ ions in water produced copper(I)–bis(pyridylimine) **1** (Figure 1a) as the uniquely observed product. Aqueous solutions of **1** are stable under anaerobic conditions. The stabilization of imine ligands and Cu^I^ is mutual and cooperative, as neither imine nor Cu^I^ is stable in water in the absence of the other.

The incorporation of 2,9-diformylphenanthroline allowed the formation of an analogous dicopper(I) helicate **2** (Figure 1b), which could be further elongated to a tricopper(I) helicate by replacing primary amines with 8-aminoquinoline.^44^ We explored the effects of solubility and solvent on helicate formation. Neutral primary amines produced helicates in acetonitrile, while anionic sulfonated amines facilitated the formation of water-soluble helicates in an aqueous solution. These preliminary results indicated that Cu^I^ species are stable in both organic and aqueous media, providing a wide solvent tolerance for different applications and requirements.

We envisaged that the principles leading to these mono- or dinuclear complexes could be extended to produce one-dimensional metallopolymers by using diamines and dialdehydes instead of primary amines and 2-formylpyridine. The preparation of Cu^I^-based metallopolymers with different coordination motifs was explored in our group. Single-stranded polymer **3** resulted from the combination of benzidine, [3,3′-bipyridine]-6,6′-dicarbaldehyde, a phosphine ligand, and Cu^I^ (Figure 1c).^45^ Whereas covalent polymers usually show a gel-to-sol transition with increasing temperature, **3** exhibits a reverse sol-to-gel transformation upon heating, as its strands cross-link by ligand exchange at high temperatures. The ligand exchange happens at high temperature, during which two heteroleptic [CuN_2_P_2_]^+^ species form one cross-linking site [CuN_4_]^+^ and one free mononuclear [CuP_4_]^+^ that increases the entropy. This ligand exchange and [CuN_4_]^+^ cross-linker is not favored at room temperature but becomes favorable as the temperature rises due to the entropy increase. Further studies employing a bidentate phosphine ligand demonstrated photoluminescence and electroluminescence for the analogous single-strand polymer in the near-infrared region. For the single-strand polymer, we observe its emission at room temperature, at 525 and 673 nm, which are ascribed to the ligand π–π* transition and the metal-to-ligand charge-transfer band. The heteroleptic [CuN_2_P_2_]^+^ is luminescent, whereas [CuP_4_]^+^ and [CuN_4_]^+^ are less emissive. The structural change upon heating creates more less-emissive species. Therefore, upon ligand exchange, we have observed the emission decrease and color change. More complex double-helical polymers with redox and electrochemical properties were also prepared by using helicates similar to those of **2** (Figure 1b) as the repeating motif. We developed two approaches, designated “AA-BB”,^43^ where equimolar amounts of diamine and dialdehyde were employed, and “AB”,^46^ using subcomponents integrating both amino and aldehyde groups, to synthesize different double stranded polymers. Control over polymer length, regioselectivity, and stereoselectivity have been explored in detail.^46,47^ Our work on Cu^I^-templated double-helical polymers has been summarized elsewhere.^48^

Our early work on generating three-dimensional cages incorporating Cu^I^ showed that the reaction between an elongated tetraamine subcomponent, 6-methyl-2-formylpyridine, and Cu^I^ affords a tubular Cu^I^_8_L_4_ structure **4** (Figure 1d) that binds linear metalloorganic guests.^49^ The tubular cavity of **4** is optimally filled with an Cu(Au(CN)_2_)_2_^–^ anion upon the addition of Au(CN)_2_^–^. This complex anion, however, does not form from Au(CN)_2_^–^ and Cu^I^ in the absence of the host. Host **4** thus acts to stabilize Cu(Au(CN)_2_)_2_^–^.

The stability of Cu^I^ complexes in different media, rich photophysical and electrochemical properties, and guest binding ability thus encouraged us to explore three-dimensional Cu^I^ assemblies with greater complexity.

## Ligand Engineering for Higher-Order Copper(I) Assemblies

Unlike the rigid octahedral coordination geometry of Fe^II^, for example, tetrahedral Cu^I^ is more flexible and can distort to optimize other noncovalent interactions. Its coordination environment is saturated by two pyridyl-imine ligands, thus requiring ligands with more than two such binding sites to obtain three-dimensional assemblies rather than simple grids. Moreover, the flexibility of Cu^I^ coordination can lead to lower-nuclearity assemblies instead of well-defined cages. Therefore, the judicious choice of precursors is crucial for obtaining higher-order Cu^I^ assemblies. Introducing more dispersion interactions, aromatic stacking, and steric interactions from delicate ligand design can provide new Cu^I^ cages beyond conventional grids and helicates.

### Curved Corannulene Subcomponent Drives the Formation of a *S*_10_-Symmetric [2]Catenane

**Figure 2:**
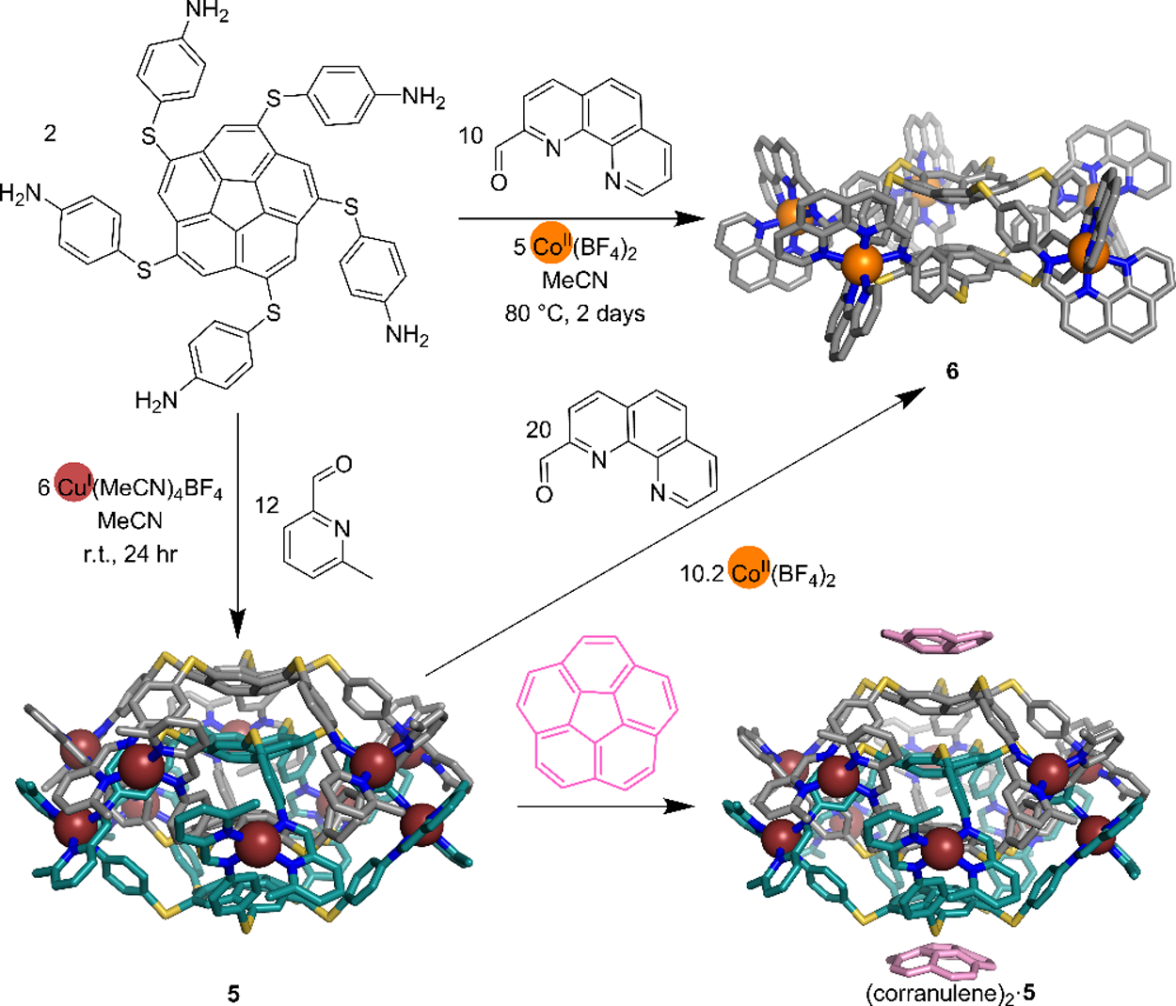
Synthesis and crystal structures of Cu^I^_10_L_2_ [2]catenane **5**, Co^II^_5_L_2_ cage **6**, and (corranulene)_2_·**5**.^1^

Aromatic stacking between ligands is useful for the synthesis of mechanically interlocked molecules.^50^ We found that the incorporation of a 5-fold symmetric corannulene subcomponent enabled the formation of a 5-fold interlocked [2]catenane, extending the family of interlocked cages from 3- and 4-fold entanglements to higher complexity.^1^ Corannulene was originally chosen as a potential 5-fold symmetry axis for dodecahedral capsules,^51^ but its curved aromatic surface instead favored smaller, stacked architectures. The reaction between the corannulene pentamine shown in Figure 2 with 2-formyl-6-methylpyridine and Cu^I^ produced a single Cu^I^_10_L_4_ product **5** with two distinct ligand environments in the ^1^H NMR spectrum. Single-crystal X-ray analysis revealed a pair of Cu^I^_5_L_2_ cages with 5-fold interlocked geometry and noncrystallographic *S*_10_ point symmetry. The distance between the mean planes of the two corannulene central rings in a single cage is 6.93(1) Å, providing sufficient space to encapsulate one corannulene from the other cage, forming a [2]catenane with bowl-in-bowl substructures. The stacked corannulene moieties are separated by a distance of 3.69(1) Å.

The formation of the interlocked structure rather than a single cage is ascribed to the van der Waals interactions arising from the substantial contact area between adjacent corannulene cores. Density functional theory (DFT) calculations in acetonitrile provided a favorable catenation energy of −272.2 kcal/mol. If dispersion effects are turned off, then catenation is predicted to be unfavorable by +42 kcal/mol, indicating the contacts induced by catenation drive the formation of this structure.

The bowl shape of the externally facing corannulenes of **5** enables interaction with further corannulene molecules in both solution and the solid state. Single crystal X-ray analysis reveals two *exo*-binding corannulene guests stacking on the corannulene ligands with a distance of 3.71(1) Å, which is very close to the separation between corannulene moieties in **5** (Figure 2).

Changing the pyridyl-imine ligand and Cu^I^ to a tridentate phenanthroline-imine ligand and an octahedral metal template did not give the same [2]catenane. Instead, a single set of resonances was observed in the ^1^H NMR spectrum, consistent with the formation of a noninterlocked M_5_L_2_ cage framework (M = Co^II^, Zn^II^), **6**.

The formation of *D*_5_-symmetric **6** was confirmed by X-ray crystallography (Figure 2). To compare the relative stabilities of the Cu^I^ and Co^II^ complexes, we added Co^II^ and 2-formyl-1,10-phenanthroline into a solution of **5**. Upon heating, a structural transformation from **5** to **6** was observed. We inferred that the iminophenanthroline-Co^II^ coordination is more enthalpically favored, as the combination of tridentate ligands and Co^II^ offers stronger coordination bonds^1^ compared to bidentate ligands and Cu^I^. The entropy also increases during this transformation, as one molecule of [2]catenane is converted to two Co^II^_5_L_2_ cages.

### Three-Dimensional Triptycene Subcomponent Generates Cu^I^_12_L_8_ Assemblies

**Figure 3:**
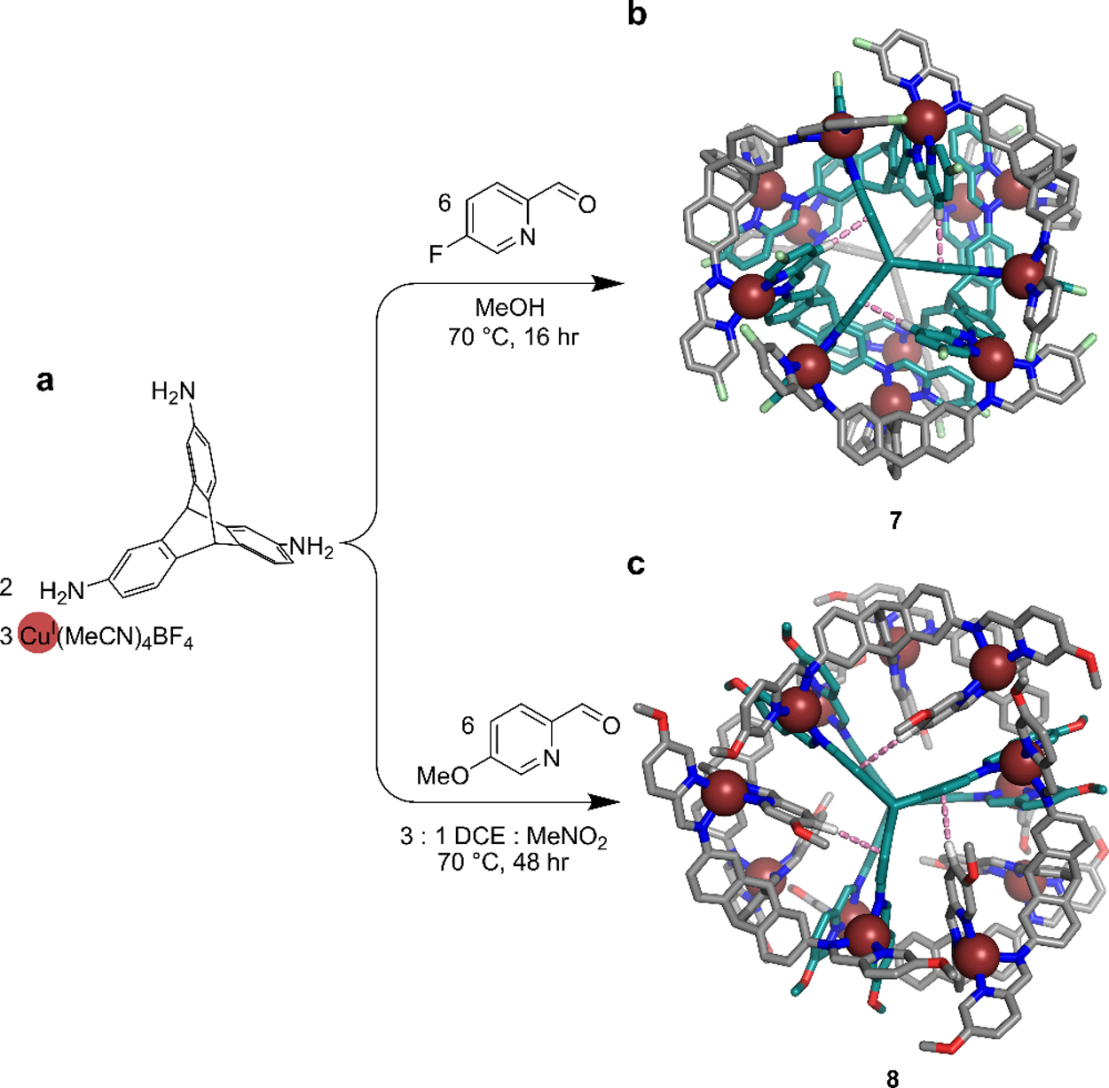
(a) Synthesis of two Cu^I^_12_L_8_ structures formed in different solvents from a triptycene subcomponent with differently substituted formylpyridines.^2^ (b) Crystal structure of *T*-symmetric double octahedral cage **7**. The carbon atoms of the outer and inner ligands are colored gray and teal, respectively. Three of the 12 C–H···π interactions between ligands are shown by pink dashed lines. (c) Crystal structure of *D*_3_-symmetric distorted hexagonal prism **8**. The carbon atoms of the equatorial and axial ligands are colored gray and teal, respectively. Three of the six C–H···π interactions between ligands are shown by pink dashed lines.

In contrast to the corannulene-based subcomponent that favors aromatic stacking, we also explored subcomponents designed to inhibit such stacking. The subcomponent 2,7,14-triaminotriptycene has curvature and rigidity with the C–H groups at the 1,8,13-positions and the triptycene bridgehead poised to provide additional steric hindrance to prevent the face-to-face stacking of ligands during subcomponent self-assembly. We hypothesized that the internal volume of assemblies formed from this subcomponent would increase to compensate for the steric clash, yielding larger architectures with novel frameworks. Initial trials using 2,7,14-triaminotriptycene as a subcomponent did indeed afford two distinct Cu^I^_12_L_8_ assemblies under different conditions (Figure 3a).^2^

The assembly of 2,7,14-triaminotriptycene, 5-fluoro-2-formylpyridine, and Cu^I^ in methanol gave a *T*-symmetric double-octahedral Cu^I^_12_L_8_ assembly (**7**). We infer that solvophobic effects play an important role in stabilizing the superstructure as the triptycene ligand exhibits poor solubility in this solvent. The high polarity of methanol drives the formation of **7** by favoring van der Waals stacking interactions between its building blocks. The crystal structure of **7** reveals 12 identical Cu^I^ vertices and two distinct ligand environments, facing the outside and inside, resulting in two sets of resonances in the ^1^H and ^19^F NMR spectra. The assembly is stabilized by 12 C–H···π interactions with an average distance of 2.66 ± 0.04 Å between pyridine and triptycene C–H groups and rings (Figure 3b).

The structural complexity of **7** led to a complex anion-binding behavior for its four distinct cavities. Four encapsulated BF_4_^–^ anions are bound in slow exchange in solution, while the larger triflate anion is bound less strongly, as inferred from the low ^19^F NMR signal intensity for bound TfO^–^. Surprisingly, the progressive addition of BF_4_^–^ to the triflate salt of **7** causes an initial increase in the signal intensity of the bound TfO^–^ together with an increase in the signal intensity of bound BF_4_^–^, indicating cooperative binding behavior. Upon binding fewer than four BF_4_^–^ anions, the remaining empty cavity expands to adapt to the larger TfO^–^ anion in an allosteric amplification of triflate binding. Addition of more than six equivalents of BF_4_^–^ disfavors the binding of TfO^–^, resulting in an increasing intensity of the signals corresponding to bound BF_4_^–^ and decreasing bound TfO^–^ signal intensity, with the system entering into a competitive binding regime. The change in the binding mode reflects the flexibility of Cu^I^ cages and their unique host–guest chemistry.

Changing the subcomponent from 5-fluoro-2-formylpyridine to 5-methoxy-2-formylpyridine and the solvent from methanol to nitromethane/1,2-dichloroethane (1:3, v/v) resulted in the formation of distinct *D*_3_-symmetric distorted-hexagonal prismatic Cu^I^_12_L_8_ assembly **8**. A more complex ^1^H NMR spectrum was observed compared to that of **7** with four sets of magnetically distinct environments for the ligands consistent with the lower symmetry of **8**. Single crystal X-ray analysis revealed that not only C–H···aryl but also stacking interactions contribute to the stability of **8** (Figure 3c). Such arene stacking, promoted by the electron donating methoxy substituent on formylpyridine, provides a driving force for the formation of compound **8**. The presence of electron-withdrawing fluorine substituents disfavors arene stacking. The dynamic nature of the Cu^I^ cages was probed by structural transformations between cage frameworks **7** and **8**. Substituent and solvent mismatches generate mixtures of the two cage frameworks, while the pure cages can be reformed by changing the solvent.

## Vertex Engineering for Increased Complexity

Coordination cages usually employ monometallic vertices whether they are prepared via subcomponent self-assembly or from preformed organic ligands. Design principles for monometallic vertices have now been established.^52−55^ Straightforward structural control during assembly can be achieved using the well-understood coordination geometry of the metal template, together with rigid organic linkers. The incorporation of bimetallic or clustered vertices is more synthetically challenging, with such complex structures offering the potential for structural diversity, different functionality, and reactivity. Compared with monometallic vertices, bimetallic or clustered corners can have more complicated coordination geometries, rendering the assembly less predictable. A common method to synthesize such complex cages involves stepwise reactions, whereby kinetically inert metallic building blocks are first prepared and purified and then used to fabricate cages with more dynamic coordination.^56,57^

### Four-Component Self-Assembly for Dicopper(I) Helicate-Bridged Cages

**Figure 4:**
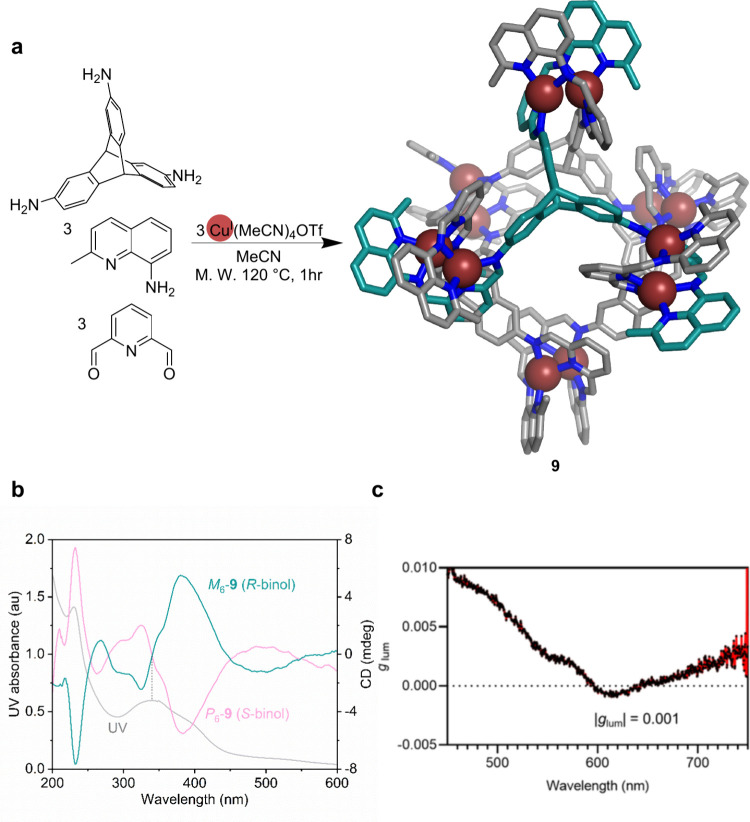
(a) Synthesis and DFT-optimized structure of Cu^I^_12_L_4_. The carbon atoms of one ligand are colored turquoise. (b) UV/vis and CD spectra of *P*_6_-**9** and *M*_6_-**9**. (c) Photoluminescence spectrum of **9**.^3^

We recently showed that Cu^I^ cages with homobimetallic corners could be synthesized in one step through a subcomponent self-assembly involving three distinct subcomponents. The reaction between 2,7,14-triaminotriptycene, 8-aminoquinaldine, 2,6-diformylpyridine, and Cu^I^ yielded Cu^I^_12_L_4_ cage **9** as a single product, integrating six dicopper(I) helicates at the corners of a pseudo-octahedron (Figure 4a).^3^ Only one set of ligand proton signals was observed in the ^1^H NMR spectrum, demonstrating high symmetry. The geometry of this Cu^I^ cage could not be produced using planar triamines, suggesting that the curvature of triptycene plays an important role in the formation of the closed structure.

The vertex design of **9** was based on prior work, where the reaction between aniline, 8-aminoquinaldine, 2,6-diformylpyridine, and Cu^I^ generated two kinds of dicopper(I) helicate with either head-to-head or head-to-tail conformations.^46^ When we used 2,7,14-triaminotriptycene as the aniline, only head-to-tail dicopper(I) helicate junctions were formed based on 2D NMR analysis. We infer that self-sorting happens during self-assembly, where side products containing the head-to-head helicate configuration transform into a head-to-tail configuration to form the closed cage. Moreover, in the presence of the diformyl compound and two amines, the system still forms a cage instead of oligomers.

The dicopper(I) helicate corners expand the cavity of the cage. Its volume is 344 Å^3^, which is large considering the short (ca. 8.8 Å) distance between amino groups in the subcomponent. Its cavity binds 1-hexylsulfonate as well as perfluoro-1-hexanesulfonate, a pollutant that has been the subject of recent attention,^58^ with their carbon chains threading into the cavity. In contrast, the larger anion tetraphenylborate interacts with **9** only weakly in its peripheral windows.

The stereochemistry of self-assembled structures enables applications in chiral recognition,^7,59^ catalysis,^60−62^ and chiroptics.^63^ The chirality of metal–organic cages is often a consequence of ligand chiral centers and the Δ vs Λ handedness of metal vertices.^7,64,65^ Incorporating dicopper(I) vertices into **9** provides another, less studied, chiral moiety: the *P* vs *M* helicity of helicates.^47^ We found that the helicity of the six dicopper(I) helicates could be controlled by the addition of the chiral ligand 1,1′-bi-2-naphthol (BINOL) during self-assembly. The use of (*S*)-BINOL as an additive enhances the proportion of *P*_6_-**9**, while (*R*)-BINOL favors *M*_6_-**9** (Figure 4b). More detailed explorations, however, indicate that added BINOL does not change the ratio between *P*_6_-**9** and *M*_6_-**9** after cage preparation, as it does not appear to interact with the cage, as probed by ^1^H NMR spectroscopy. We thus infer that BINOL coordinates to the Cu^I^ template initially, forming a chiral intermediate that determines the helicate stereochemistry after ligand exchange with the stronger iminopyridyl and iminoquinolyl ligand strands.

Circular dichroism spectra of the chiral cages prepared at higher temperatures exhibit decreasing Cotton effects, which is also consistent with our proposed mechanism, since the BINOL-Cu^I^ complex disassembles at higher temperatures.

Benefiting from the closed-shell nature of the Cu^I^ ion, cage **9** has a red emission centered at around 600 nm. When enantiopure BINOL was added during assembly, the helicity-biased cage displayed circularly polarized luminescence with a dissymmetry factor of 0.001 (Figure 4c).

### Phosphino(pyridine) Subcomponent for Homobimetallic and Heterobimetallic Vertices

**Figure 5:**
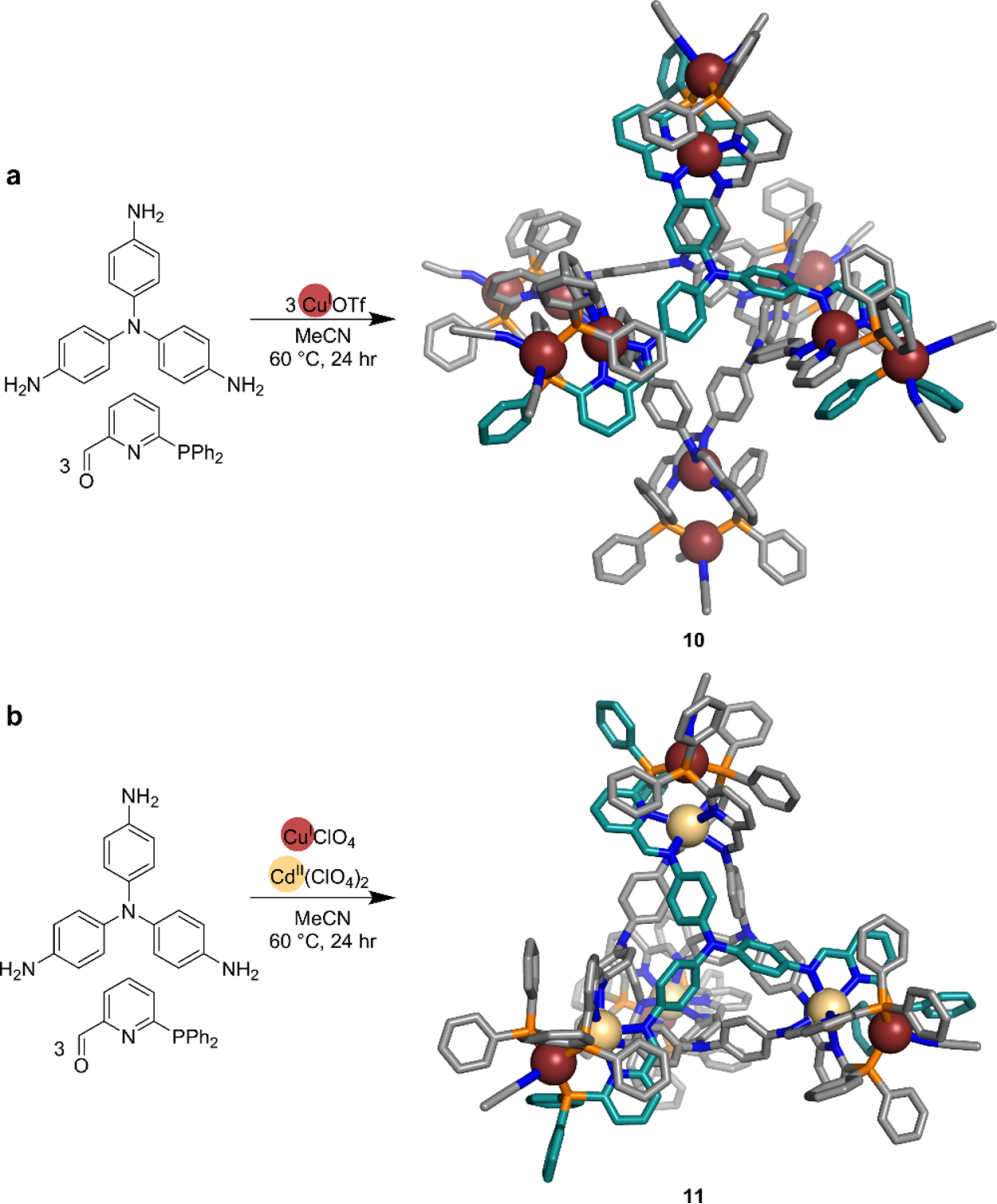
(a) Synthesis and crystal structure of Cu^I^_12_L_4_ cage **10**. (b) Synthesis and crystal structure of Cu^I^_4_Cd^II^_4_L_4_ cage **11**. Copper ions are brown, and cadmium ions are cream. For **10** and **11**, the carbon atoms of one ligand are turquoise and the rest are gray.

The introduction of ligands containing both soft and hard donor atoms has enabled the construction of cages with bimetallic vertices. Our attempts yielded Cu^I^_12_L_4_ cage **10**, incorporating the above-mentioned phosphino subcomponent, tris(4-aminophenyl)amine, and Cu^I^ (Figure 5a).^4^ The crystal structure of **10** revealed a high-symmetry pseudo-octahedron composed of six identical dicopper(I) vertices. The dicopper(I) complexes in the cage adopt a head-to-head conformation to form a closed cage, in contrast with the head-to-tail configuration prepared from monoanilines.^66^ In each dicopper(I) vertex, the innermost Cu^I^ is chelated by two pyridyl-imine motifs, as with the previously discussed Cu^I^-based cages, but the coordination is distorted from a regular tetrahedral coordination geometry. The outermost Cu^I^ ions are each coordinated by two phosphorus atoms together with two acetonitrile molecules, completing their tetrahedral coordination geometry. The diverging coordination vectors of the ligand readily bridge pairs of metal centers, thus enforcing bimetallic coordination motif formation.

An Ag^I^_12_L_4_ cage analogous to **10** was obtained when Ag^I^ was used in place of Cu^I^, allowing the two environments to be probed by multinuclear NMR experiments. Both ^1^H-^109^Ag and ^31^P-^109^Ag couplings were observed in the NMR spectra, allowing the signals of each magnetically distinct Ag atom to be identified and providing proof that the head-to-head configuration was maintained in solution.

The cavity volume of **10** is only 90 Å^3^, smaller than that of **9** (344 Å^3^), although both contain dicopper(I) vertices bridged by similar-sized tritopic ligands. The distinct cavity sizes may be ascribed to the different conformations of the vertices. Cage **9** contains head-to-tail helicates, which act as longer bridges between the two tritopic ligands. The distances between ligands are shorter in **10**, where the helicate corner adopts a head-to-head conformation. Such dicopper(I) helicate-bridged cages may thus potentially exhibit adjustable cavity sizes, if the vertex configuration can be controlled.

The different coordination environments presented by the iminophosphino ligands incorporated into **10** encouraged us to explore their potential in the fabrication of cages containing heterobimetallic vertices. When metal ions with different coordination preferences, such as softer tetrahedral metals and harder octahedral metals, were combined, discrimination was observed between the N-binding and P-binding sites. Combination of Cu^I^ and Cd^II^ in an equimolar ratio yields face-capped tetrahedral cage **11** containing heterobimetallic vertices (Figure 5b). Each vertex of this cage incorporates an inner Cd^II^ coordinated by six nitrogen atoms and an outer Cu^I^ bound to three phosphorus donors and one acetonitrile. The chelation of Cd^II^ by pyridyl-imine units brings together three ligands at each vertex, leading to three phosphorus donors arranged in an approximately tetrahedral geometry suitable for binding an extra Cu^I^. The solvent acetonitrile then saturates the fourth coordination site of Cu^I^. Incorporation of both kinds of coordination sites is essential in the selective formation of this cage. Although both metal ions are classed as soft acids, the more highly charged Cd^II^ centers are harder than the lower-charged Cu^I^, thus driving selective coordination at the pyridyl-imine sites and leaving the softer phosphorus donors free to bind Cu^I^. The method was also applied to obtain a larger Cu^I^_4_Cd^II^_4_L_4_ cage with more scope for guest binding, which is necessary for the development of applications. Our strategy to produce cages with well-defined heterobimetallic vertices thus compliments other heterometallic cage strategies, where different metal ions are incorporated in different coordination sites within the structure.^67−69^

## Guest-Controlled Self-Assembly of Cu^I^ Cages

**Figure 6:**
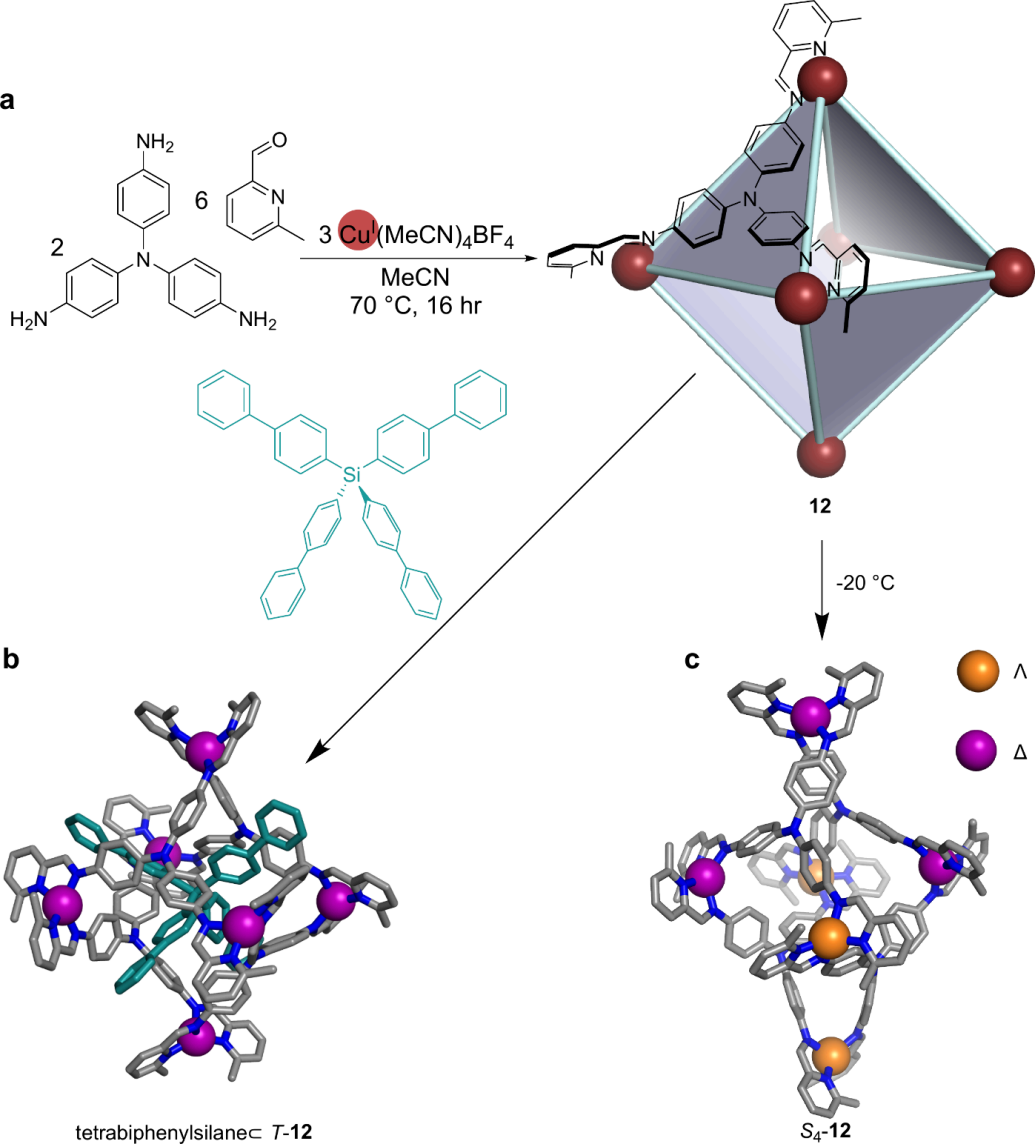
(a) Synthesis of cage **12** as a mixture of diastereomers. (b) Synthesis and crystal structure of suit[4]ane tetrabiphenylsilane⊂*T*-**12**, with the guest colored turquoise. (c) Crystal structure of *S*_4_-symmetric **12**, the diastereomer observed to predominate in solution at −20 °C.^5^

We have also developed a guest-induced structural reconfiguration in a Cu^I^ cage. Tris(4-aminophenyl)amine, 6-methyl-2-formylpyridine, and Cu^I^ generated different diastereoisomers of Cu^I^_6_L_4_ pseudo-octahedral cage **12** (Figure 6a).^5^ Reducing the temperature to 253 K resulted in a single set of sharp, 3-fold desymmetrized signals in the NMR spectrum, consistent with the formation of an *S*_4_-symmetric structure (Figure 6c). The solid-state structure of *S*_4_-**12** was unambiguously assigned through single crystal X-ray diffraction. Dissolution in DMSO favored the *T*-**12** and *S*_4_-**12** diastereomers, as confirmed by NMR spectroscopy. The mixture of room-temperature diastereomers of **12** formed contrasts with the situation with **10**, which formed a single diastereomer at room temperature. Cage **12** also differs from the Zn^II^_6_L_4_ pseudo-octahedral cage reported with the same trianiline subcomponent,^70^ but where Cu^I^ was replaced by Zn^II^ and 6-methyl-2-formylpyridine was replaced by 2-formylphenanthroline. Only a single set of signals was observed in the ^1^H NMR spectrum of the Zn^II^_6_L_4_ cage, indicating that only the highest-symmetry structure with homochiral metal centers was present. The single crystal structure of Zn^II^_6_L_4_ confirmed its homochiral configuration. Both Cu^I^ and Zn^II^ have a d^10^ electronic configuration, suggesting that both structures may have a flexible coordination geometry due to a lack of ligand field stabilization energy. The tetrahedral coordination geometry of Cu^I^, however, may endow the coordination junctions with more flexibility due to less steric hindrance, leading to smaller energy differences between the diastereomers.

Addition of the tetrahedral guests tetraphenylborate or tetraphenylmethane induced the rearrangement of the Cu^I^_6_L_4_ mixture of diastereomers to form *T*-Cu^I^_6_L_4_ selectively, with one equivalent of guest bound inside. This guest binding behavior contrasts with that seen by cages formed with the same trianiline subcomponent but utilizing octahedral Zn^II^ and Cd^II^ ions and 2-formylphenanthroline.^70^ These M^II^_6_L_4_ capsules were found to bind tetraphenylborate derivatives peripherally in solution across one of the apertures of the cage, while tetraphenylmethane did not bind. We hypothesize that the difference in the binding behavior between **12** and its Zn^II^ and Cd^II^ analogues may arise from two factors. First, the lower coordination number of Cu^I^ requires fewer Cu^I^–N bonds to be broken and reformed for guest encapsulation. Second, the softer Cu^I^ ions are expected to form weaker coordination bonds to nitrogen donors than the harder Zn^II^ and Cd^II^ ions; thus, the Cu^I^–N coordination bonds show higher lability, allowing **12** to more readily disassemble and reassemble around the guests.

When tetrabiphenylsilane, a tetrahedral guest with elongated arms, was added to **12**, a host−guest complex was formed between *T*-symmetric **12** and tetrabiphenylsilane (Figure 6b). NMR and MS data corroborated the formation of the structure, and single crystal X-ray data showed the formation of a mechanically interlocked suit[4]ane. Suit[4]anes are a class of mechanically interlocked molecules, first synthesized by Stoddart and co-workers,^71^ where a guest with multiple arms protrudes through the pores of a polycyclic host, resulting in the formation of a mechanical bond.^72^

Previous examples of suitanes used only planar guests to form flat suitanes.^73^ Tetrabiphenylsilane⊂*T*-**12** was thus the first report of a suit[n]ane with a nonplanar axle.

Fluorinated steroids were also found to interact with **12** at low temperatures, favoring the formation of *T*-**12**. Notably, no interaction was observed for the analogous nonfluorinated steroids. ^1^H–^19^F HOESY experiments revealed correlations between the fluorine atom on the steroid and the phenyl core of the *T*-**12**. This interaction was still observed when the cavity was blocked by tetrabiphenylsilane, suggesting an external binding mode.

## Conclusions and Pespectives

The use of Cu^I^ in subcomponent self-assembly has attracted less attention than using octahedral metal ions,^19,20,22,25^ although it has shown great potential for the generation of novel structures. Cu^I^ cages with intricate structures can be prepared through the rational incorporation of the subcomponents,^1,2^ bimetallic vertices,^3,4^ and guests^5^ discussed in this Account. Manipulating aromatic stacking can generate Cu^I^ cages with diverse topologies and geometries.^1,2^ The different Cu^I^ cages exhibit distinct guest binding properties. The incorporation of dicopper(I) helicates paves the way for the enantioselective preparation of these cages.^3^ This ability may lead to the creation of new technologically relevant species that can emit circularly polarized light upon photoexcitation. The dynamic nature of Cu^I^ coordination facilitates structural transformations under the action of different stimuli, such as temperature, solvent change, and the addition of a guest.^5^ Fundamental studies of these processes can help us understand how noncovalent interactions help dictate the formation of complex structures, as often happens in biological systems.

Future developments associated with Cu^I^ cages relate to the construction of more useful and predictable host–guest complexations and tuning their photophysical and electronic properties. We hope that the synthesis of novel Cu^I^ cages and the exploration of their functions will benefit from the design principles and guest-responsive behavior outlined in this Account. Moreover, the redox behavior and photoluminescence of Cu^I^ can enable applications in the fields of light emitting devices and resistive switches.^74^ The redox behavior of Cu^I^ also holds potential in catalysis, where Cu^I^ cages could serve as confined reaction environments, enhancing the selectivity and efficiency of a reaction. The Cu^I^ vertices might facilitate electron transfer, helping to enable photoredox catalysis and other transformations requiring controlled oxidation and reduction. Another potential application is environmental remediation, where a Cu^I^ cage may capture a pollutant and oxidatively degrade it through a photoinitiated process. Cu^I^ complexes with pyridyl-imine ligands can possess good stability in water, in contrast with Zn^II^- or Cd^II^-based structures.^75^ Cu^I^ cages may thus become suitable agents for drug delivery and bioimaging thanks to their photoluminescence. However, challenges in cage stability, solubility, and biocompatibility should also be considered in the context of practical applications. Cu^I^ cages tend not to be as stable as those incorporating octahedral first-row transition metals, although using sterically hindered ligands, encapsulation by amphiphilic polymers, or hybridization with inorganic porous materials would enhance stability. By utilizing different anions, Cu^I^ cages can be tuned to be hydrophilic or hydrophobic, facilitating guest binding in different media. Lastly, biocompatibility must be considered for biomedical applications. While copper is an essential trace element, excessive Cu^I^ exposure can be cytotoxic. Encapsulating a cage in a biocompatible nanocarrier might help to minimize metal leakage. We anticipate that, by using Cu^I^ cage-based host–guest chemistry, new functions in aqueous media such as chemical purification, catalysis, and theranostics can be achieved to address practical problems relating to the environment, industry, and biomedicine.
